# Advance care planning and the parental geographical background in pediatric palliative home care: a retrospective chart review

**DOI:** 10.1007/s00431-022-04469-w

**Published:** 2022-05-04

**Authors:** Lars Dinkelbach, Maren Galushko, Anne Oommen-Halbach, Melisa Felek, Oliver Dechert, Laura Trocan, Gisela Janßen

**Affiliations:** 1 Department of Pediatric Oncology, Hematology and Clinical Immunology, Centre for Child and Adolescent Health, Medical Faculty, Heinrich-Heine-University, Duesseldorf, Germany; 2 Centre for Child and Adolescent Health, HELIOS Klinikum Krefeld, Lutherplatz 40, 47805 Krefeld, Germany; 3 Department of the History, Philosophy and Ethics of Medicine, Medical Faculty, Heinrich-Heine-University, Duesseldorf, Germany

**Keywords:** Pediatrics, Palliative care, Medical orders for life sustaining treatment, Advance care planning, Cultural background, Culturally competent care

## Abstract

The relevance to acknowledge the parental migration history in pediatric palliative care is widely recognized. However, its influence on integral parts of advance care planning (ACP) is unknown. In this non-interventional cohort study, we aimed at identifying systematic differences between pediatric palliative patients with varying parental countries of origin regarding medical orders for life-sustaining treatment and the location of patients’ death. Two hundred eighty-eight pediatric cases in an ambulant pediatric palliative care setting in Germany were retrospectively analyzed using multinomial logistic regression models. Agreements on medical orders for life-sustaining treatment (MOLST) differed significantly between patients with varying parental countries of origin. Full code orders for life-sustaining treatment were made more often in Turkish families than in German families. There were no significant associations between the patients’ location of death and the parental countries of origin. However, confounder-analysis revealed a strong association between the patients’ underlying disease and the orders for life-sustaining treatment as well as the location of death.

*Conclusions*: Even this study indicates that the parental geographical background as an important sociocultural aspect might have an impact on ACP decisions for children and adolescents with life-limiting conditions, other factors as the patients’ underlying disease can be more crucial for decision making in pediatric palliative care. The reason for the differences found might lay in cultural preferences or barriers to appropriate care. The inclusion of sociocultural aspects in decision-making is crucial to guarantee culture-sensitive, patient-centered pediatric palliative care.

**Supplementary information:**

The online version contains supplementary material available at 10.1007/s00431-022-04469-w.

**Table:** 

**What is Known:** *• Decision making processes in pediatric palliative care are influenced by various cultural and religious norms*. *• So far, there is little knowledge about tangible differences with regard to end-of-life care decisions according to the geographical background of parents*.	
**What is New:** *• End-of-life care decisions varied significantly with the parental country of origin*. *• Other factors as the underlying disease may be more crucial for decision making in pediatric palliative care*.	

## Introduction

In pediatric palliative care, advance care planning (ACP) involves determining the framework of end-of-life care based on medical needs and the preferences of affected children and their parents [1]. Implementation of ACP interventions in pediatric palliative care (PPC) have been shown to increase the perceived quality of life during end-of-life [2]. As part of the ACP process, medical interventions in the light of a possible future clinical deteriorations should be discussed and preferences regarding medical orders for life-sustaining treatment (MOLST) should be documented. These decisions directly influence care endpoints such as the patients’ place of death [3].

The family sociocultural background involves a cross-generational transmitted variety of values, religious beliefs, and attitudes which have a significant impact on how individuals cope with experiences of illness and death [4]. These cultural imprints can lead to a varying acceptance and content of ACP discussions in adult and pediatric palliative care [5–7]. The acknowledgment of sociocultural preferences in ACP is important for caregivers to provide culture-sensitive, patient-centered care [7, 8]. In order to improve cultural coherence of decision-making in ACP in minority families, this study aims to identify similarities and differences about MOLST and the patients’ location of death according to the geographical background of parents in pediatric palliative home care. In doing so, we examine the individual migration history as one aspect of the sociocultural familial background to better understand the factors influencing the ACP process.

## Methods

Between January 2013 and December 2018, 304 patients received specialized palliative care by the pediatric palliative home care team “Sternenboot” of the University Hospital Düsseldorf, Germany. In total, 293 of these cases (96.4%) had available information on the parental geographical background. Four patients (1.3%) were taken care for by foster parents, and in one patient, the location of care was unknown. These five cases were excluded from further analysis, leading to remaining 288 cases included in the study.

Information on patients’ parental country of origin (including second generation descendants) as documented during patients’ initial admission, clinical characteristics, and if applicable, the patients’ location of death were retrieved from patients’ medical records. For statistical analyses, the included patients were categorized in four groups based on the demographic distribution in the study population: German families, Turkish families, families from countries with an Arab majority, and families with another or mixed geographical background (see Supplemental Table 1).

Medical orders for life-sustaining treatment (MOLST) were documented by pediatricians and nurses following the discussion of preferences with the patients’ parents (or their legal guardians, if applicable). Involvement of the affected child or adolescent was considered whenever possible. During ACP discussions, the patient’s current medical state and prognosis was addressed. Possible medical interventions in the light of potential clinical deterioration were explained and the consequences for the patient and his/her prognosis was discussed. The families’ and/or patients’ preferences for or against medical interventions in emergency situations were documented in a structured way using a previously published MOLST form [9]. In the case of language barriers, interpreters were recruited. Where no professional interpreter was available, interpreters out of the patient’s family environment or medical staff were recruited to ensure that all families could follow the discussions and comprehend the implications of the decisions made. The MOLST discussions and decisions were renewed every 3–12 months depending on child’s health status. For statistical analysis, the last documented agreement was used. Agreements on medical orders for life-sustaining treatment were categorized in Do Not Attempt Cardio-Pulmonary Resuscitation (DNACPR) orders if agreements included comfort care (including nasopharyngeal suctioning) only, Treatment Limitations (TL) if agreements included some but not all measures of life sustaining treatments (e.g., non-invasive ventilation) and Full Code (FC) if agreements on medical orders for life-sustaining treatment at least included cardio-pulmonary resuscitation, cardiac massage, and intubation (or tracheostoma).

To identify possible differences concerning agreements on medical orders for life-sustaining treatment (DNACPR, TL, or FC) and the location of death (at home, hospice, or hospital) as a function of varying parental origin two multinomial regression analysis were calculated. To adjust for potential confounders, patients’ disease group (oncological diseases vs. all other diseases) and the patients’ age (at the last obtained visit) as well as the patients’ location of care (both parents, one parent alone, at a nursing home or hospice) were additionally included into the regression model. In further analyses, two chi-squared tests were calculated, one to assess whether the existence of a MOLST documentation differs between families with varying geographical backgrounds. Another chi-squared test was calculated to assess whether there was a significant variation with MOLST-agreements and the patients’ place of death. *P*-values below 0.05 (two-tailed) were considered statistically significant.

## Results

In total, 288 patients (142 girls, 49.3%) were included in this single-center, retrospective study. The majority of patients’ families had a German geographical background (181; 62.8%). The most common foreign geographical background was Turkish (39; 13.5%). Twenty-four families (8.3%) originally came from countries with an Arab majority, while 44 parents (15.3%) had other countries of origin or a mixed geographical background. The median age at inclusion in palliative care was 6 years and 2 months (74 months, range 0–309 months). One hundred three patients (35.2%) suffered from oncological diseases e.g., glioblastoma, rhabdomyosarcoma, or acute myeloid leukemia. The remaining 185 patients (64.2%) suffered from either progressive conditions (e.g., leukodystrophies or mitochondriopathies) or irreversible but non-progressive conditions leading to a high likelihood of a premature death, e.g. syndromal conditions, perinatal hypoxia, or intraventricular hemorrhage. Details on demographics are given in Table 1.

**Table 1:** Demographic data of pediatric patients who received palliative care between January 2013 and December 2018. This table depicts the demographic and clinical characteristics of the included patients, divided by their geographical backgrounds

	Total *n* = 288		German *n* = 181		Turkish *n* = 39		Countries with an Arab majority *n* = 24		Others/mixed *n* = 44	
General demographics										
Age at referral, median (range in months)	74	0–309	89	0–309	119	0–261	50	0–172	50.5	0–232
Age at last visit or death, median (range in months)	81	0–339	102	0–339	124	2–333	57	1–206	57	0–284
Gender female (%)	142	49.3%	86	47.5%	24	61.5%	16	66.7%	16	36.4%
Details on palliative care										
Duration of care, median (range in months)	4	0–84	4	0–84	10	0–80	2	0–56	3	0–82
Number of visits, median (range)	11	1–100	10	1–100	17	1–68	7	1–52	12	1–96
*Place of care*										
Both parents	199	69.1%	124	68.5%	28	71.8%	18	75.0%	29	65.9%
One parent (mother or father)	57	19.8%	38	21.0%	7	17.9%	2	8.3%	10	22.7%
Nursing home or hospice	32	11.1%	19	10.5%	4	10.3%	4	16.7%	5	11.4%
Disease categories										
Oncological	103	35.8%	64	35.4%	11	28.2%	10	41.7%	18	40.9%
Non-oncological	185	64.2%	117	64.6%	28	71.8%	14	58.3%	26	59.1%
Status of care										
Continued care	39	13.5%	30	16.6%	4	10.3%	2	8.3%	3	6.8%
Termination of care	63	21.9%	34	18.8%	10	25.6%	5	20.8%	14	31.8%
Death	186	64.6%	117	64.6%	25	64.1%	17	70.8%	27	61.4%
Median age at death (range in months)	75	0–320	94	0–320	106.0	2–282	47	1–170	61	0–236

### Medical orders for life-sustaining treatment (MOLST)

Two hundred forty (85.7%) cases had documented agreements on MOLST. The existence of a MOLST documentation did not differ with varying parental geographical backgrounds (*p* = 0.993, chi-squared test). Overall, DNACPR orders were made most frequently (*n* = 131, 54.6%) while FC orders were made in 54 of the cases (22.5%). For 55 patients (22.9%), medical orders for TL were made. In TL orders, mask ventilation in 46 cases (83.6%) or intubation in three cases (5.4%) without further life-sustaining treatment was agreed. In eight cases (14.5%) with TL orders, airway management already included a tracheostoma, and in three cases (5.4%), cardiac massage without intubation was agreed.

**Fig. 1:**
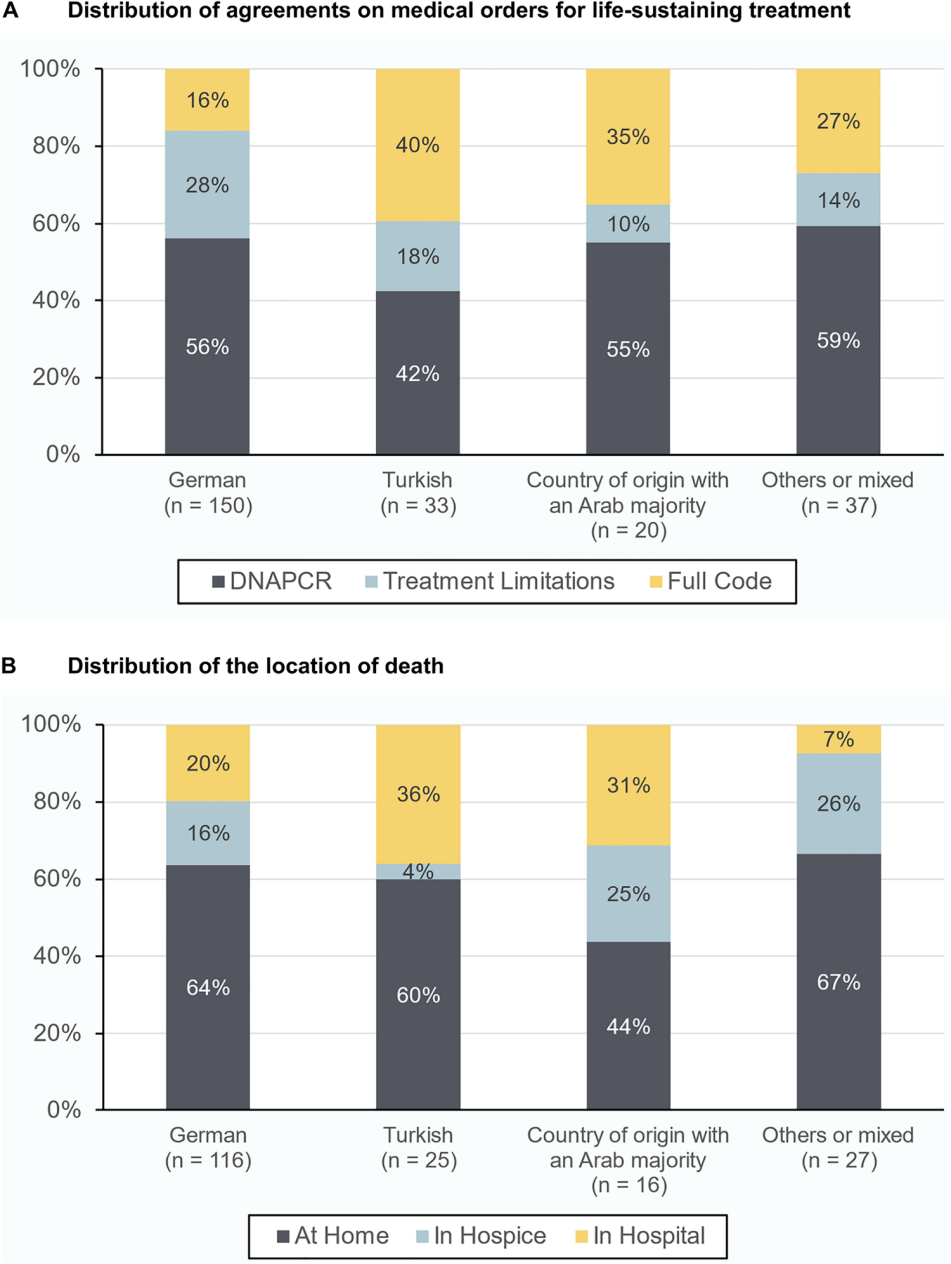
Distribution of pediatric palliative care endpoints in relation to the parental geographical background. **1A** Distribution of agreements on medical orders for life-sustaining treatment in relation to the parental geographical background. Medical orders for life-sustaining treatment were made in mutual agreement with a pediatrician of the pediatric palliative care team and the patients’ parents. These agreements were retrospectively categorized in Do Not Attempt Cardio-Pulmonary Resuscitation (DNACPR) orders if the agreements included comfort care only (including nasopharyngeal suctioning), Treatment Limitations if the agreements included some but not all measures of life sustaining treatments, and Full Code if the agreements at least included all three of the following measures: cardio-pulmonary resuscitation, cardiac massage, and intubation (or tracheostoma). **1B** Overview on the location of death of patients which received pediatric palliative care between January 2013 and December 2018. Deaths which occurred until December 2019 were included in the statistical analysis. The category “At Home” refers to the patients’ primary location of care, e.g., at their parents’ place or at a nursing home

Regardless of the parental geographical background, agreements on DNACPR were most frequent (German families: 84, 56.0%; Turkish families: 14, 42.4%; families from countries with an Arab majority: 11, 55.0%; others or mixed countries of origin: 22, 59.5%). In German families, TL orders were second most frequent (42 TL orders, 28.0%; 24 FC orders, 16.0%) while FC orders were second most frequent in Turkish families (6 TL orders, 18.2%; 13 FC orders, 39.3%), in families with a country of origin with an Arab majority (2 TL orders, 10.0%; 7 FC orders, 35.0%) and in families with others or mixed countries of origin (5 TL orders, 13.5%; 10 FC orders, 27.0%). The distribution of agreements on MOLST are illustrated in Fig. 1A.

The resulting multinomial logistic regression model for the documented agreements on medical orders for life-sustaining treatment explained 39.2% of the variance (Nagelkerkes R^2^, Χ2 = 99.4, *p* < 0.001). The documented agreements on medical orders for life-sustaining treatment differed between families with varying parental countries of origin (Χ^2^ = 13.9, *p* = 0.031). Turkish families (*OR* = 3.4; 95%*-CI*: 1.2; 9.3; *p* = 0.018) more often decided on FC orders in comparison to German families.

The documented agreements on MOLST also differed significantly with the patients’ disease groups (Χ^2^ = 81.7, *p* < 0.001). Families of patients with non-oncological diseases were more likely to agree on TL (*OR* = 63.5; 95%-*CI*: 8.4; 476.9; *p* < 0.001) or FC orders (*OR* = 21.9; 95%-*CI*: 6.3; 75.8; *p* < 0.001) in comparison to families of patients with oncological diseases. The documented agreements on MOLST did not differ significantly with the patients’ location of care Χ^2^ = 0.37, *p* = 0.985) or the patients’ age (Χ^2^ = 2.7, *p* < 0.260).

### Location of death

Of the 288 included patients, 186 patients (64.6%; 90 female) died during the observation period. The median age at death was 6 years and 3 months (75 months, range 0–320). Most patients died at home (114, 61.3%), 39 patients (21.0%) in a hospice, and 31 patients (16.7%) in a hospital. One patient died during a car ride and one patient died on holiday with friends; both patients were excluded from following analyses.

Of the remaining patients, most died at home, regardless of the parental geographical background (German families: 74, 63.8%; Turkish families: 15, 60.0%; families from countries with an Arab majority: 7, 43.8%; others or mixed countries of origin: 18, 66.7%). For details on the distribution of the location of death see Fig. 1B.

The resulting multinomial regression model for the patients’ locations of death explained 33.2% of the variance (Nagelkerkes R^2^, Χ^2^ = 60.4, *p* < 0.001). The patients’ locations of death did not vary significantly with the parental country of origin (Χ^2^ = 9.8, *p* = 0.134) or with the patients’ age (Χ^2^ = 0.273, *p* = 0.872). However, significant associations between the locations of death and the patients’ locations of care (Χ^2^ = 19.5, *p* < 0.001) as well as the patients’ disease group (oncological vs non-oncological diseases; Χ^2^ = 20.8, *p* < 0.001) were found. Patients living either at both parents (*OR* = 0.1; 95%-*CI*: 0.0; 0.3; *p* < 0.001) or at one parent (*OR* = 0.1; 95%-*CI*: 0.0; 0.6; *p* = 0.009) were less likely to decease in a hospice than patients taken care for in a nursing home or hospice. Patients suffering from non-oncological diseases were more likely to decease in a hospital (in comparison to decease at home) than patients with oncological diseases (*OR* = 7.6; 95%-*CI*: 2.9; 20.1; *p* < 0.001). The likelihood to decease in a hospice (in comparison to decease at home) did not vary significantly with the underlying disease group (*p* = 0.235).

In 157 deceased patients, information on MOLST was available. A significant variation of the MOLST and the patients’ place of death was found (Χ^2^ = 51.9, *p* < 0.001). Of 17 deceased patients with FC orders, the majority (*n* = 14, 82.4%) died in hospital, and three cases (17.6%) died at home. In patients with DNAPCR orders, at home was the most frequent place of death (*n* = 82, 74.5%); 18 patients (16.4%) died in a hospice while ten patients with DNAPCR orders (9.1%) died in a hospital. Of the 30 deceased patients with TL orders, 14 died at home (46.7%), nine in a hospital (30.0%), and seven in a hospice (23.3%).

## Discussion

### Main findings of the study

This retrospective chart review of a large, representative pediatric palliative care sample in Germany investigates the relationship between the parental geographical background and mutual agreements on medical orders for life-sustaining treatment (MOLST) or the patients’ location of death.

#### MOSLT were frequently made and the existence did not differ with parental geographical background

In 85.7% of the patients included in our study, decisions on MOLST were documented, a remarkably high percentage considering that in an US study of deceased children in only 18% of cases a MOLST was documented at the time of death, even in patients receiving pediatric palliative care [10]. The MOLST forms were completed in mutual agreement following discussions of healthcare professionals and the patients’ parents on the prognosis of the underlying condition, the advantages and potential harm of medical interventions in possible future clinical deterioration. Patients were involved as much as possible according to their state of health, maturity, and understanding of their situation. The high percentage of families with a documented medical order emphasizes the broad acceptance among parents to participate in the ACP process of their children. However, it has to be noted that the ACP process and MOLST documentation is an integral part of work of the PPC team analyzed in the current study and is frequently addressed within home care visits.

McDermott and Selman (2018) concluded that patients depending on their cultural background can differ in their acceptance of a formal, more documented ACP process versus an informal, more communication-focused one (McDermott & Selman, 2018). In contrast, our data shows that whether a MOLST was documented did not differ with varying parental geographical backgrounds which indicates the formal ACP process in our study was generally accepted, irrespective of the parental geographical background.

#### MOLST differ with varying geographical backgrounds and disease groups

In our study, MOLST in Turkish families more often included life-sustaining treatments (Full code orders) in comparison to German families. The differences found may either reflect barriers to appropriate care or cultural preferences.

Language barriers are frequent in pediatric palliative care in Germany [11] and have been shown to influence integral parts of care in pediatric patients with special care needs [12, 13]. A closer look on our data (see Fig. 1A) reveals that in comparison to German families, the higher percentages of FC orders in Turkish families at least partially were driven by a lower likelihood of TL orders in which some, but not all measures of life sustaining interventions were agreed on. Thus, language barriers in minority families might have prevented more substantial discussions with healthcare professionals and therefore prevented more individualized agreements on advance care planning processes which might have led to decisions prone to FC orders without any limitations for life sustaining treatments. During the observation period of our study, interpreters were recruited out of the family environment or medical staff to overcome language barriers if professional interpreters were not available. However, a trained professional interpreter might have improved the families’ comprehension and thus would have influenced the results. We strongly encourage the use of professional face-to-face interpreters in the case of evident language barriers if available.

Even though our data suggests a general high acceptance of ACP in our study, the formal documentation of the mutual agreements on MOLST might have interacted with cultural preferences for or against this type of advance care planning process [5]. Thus, the documentation of MOLST might have prevented minority families to decide for treatment limitations. In line with this assumption, a previous study has shown that written DNACPR orders were preferred in Northern and central Europe while orders in Turkey as well as in Brazil and Southern Europe were preferentially made verbally (Yaguchi et al., 2005). The documentation of ACP decisions is especially of interest for health care providers in order to have a guideline for clinical interventions while the ACP process itself might be of higher interest for parents [14].

In a qualitative study among Turkish and Moroccan cancer patients in the Netherlands, the preservation of hope and curative care until death were seen as features of good care [15]. These cultural preferences at least partially contradict the western ideas of palliative care with a stronger focus on quality of life rather than curative treatment or the discussion of unfavorable prognoses of underlying conditions [15]. Thus, cultural preferences for curative care rather than supportive care only might be a reason for the differences found. However, it is unclear whether these findings can be transferred to the very specific case of pediatric palliative care with its heterogenous patient collective and involvement of the whole family in decision making processes.

Besides the geographical background, a significant association of the patients’ underlying disease on MOLST was found. Families of patients with oncological diseases were less likely to agree on life sustaining treatments (Treatment Limitations or Full code orders) than families of patients with other diseases. The relevance of the diagnosis for the decision for or against DNACPR orders is understandable and has previously been shown [3]. Where in oncological diseases, the trajectories are relatively short and more predictable, and patients in other disease groups with sometimes very rare diseases have trajectories difficult to anticipate and often include relatively long periods of stable symptom load. Thus, to decide not to begin a treatment is more ambiguous for parents and healthcare professionals which might have led to the higher rate of decisions for life sustaining treatments in these diseases.

#### Place of death is not associated with geographical background but with place of care and disease groups

The patients’ location of death did not differ with the parental geographical background. Across all geographical groups, at home was the most frequent location of death. In total, a remarkably high percentage of 61.2% of patients died at home. In line, dying at home was the preferred location of death among a survey of bereaved parents of pediatric cancer patients [16]. Dying at home seems cross-culturally desired and would especially in Muslim families ease the religious duty of family visits of the dying patient [17]. In contrast, the determination of treatment limitations that comes along with home treatment contradicts the wish not to disrespect Gods decisions [17] and thus may be a cause of conflict within the family or conflict within the respective community. Therefore, it is crucial to address and accept the hesitation towards life limiting treatment decisions in different cultural contexts within the advance care planning process. Even the difference did not reach the level of significance, a trend towards a lower frequency of choices for hospices by families of Turkish origin was found. End-of-life care in hospices may not exactly match preferences of Turkish or Muslim families who in turn may benefit from a stronger family support [18].

It is not surprising that the location of death corresponds significantly with the location of care. Patients whose primary location of care was an institution, e.g., a nursing home or a hospice, were also more likely to die in a hospice than patients whose primary location of care was their parents place. Interestingly, the disease group is also connected with the place of death. That patients with oncological diseases die significantly more at home rather than in hospital can be seen as a fulfillment of the high percentage of DNACPR orders agreed on in those families and as a success of pediatric palliative home care treatment which encourages home care at the end of life. As disease trajectories of non-oncological diseases are less predictable and often complex, caregivers and professional might be unsure and tend to involve hospital care more often. In line, a significant variation of the patients’ place of death with MOLST were seen. In patients’ with DNAPCR orders, at home was the most frequent place of death while most patients without treatment limitations died in a hospital. The impact on MOLST agreements on the patients’ place of death was previously described [3].

#### Similarities in the advance care planning process might reflect shared norms and values

Taken together, it must be noted that the acceptance of MOLST in general was high and irrespective of the geographical groups assessed. The place of death in deceased patients did not differ between parental geographical backgrounds. Even though a higher likelihood of full code orders in Turkish families was found, it must be emphasized that DNACPR orders were most frequently made across all geographical groups assessed. This fact suggests a high general acceptance for the ACP process and for limitations of life sustaining treatments in pediatric patients with life limiting conditions, irrespective of the familiar geographical background. Therefore, this study indicates a broad common basis of shared norms and values regarding the advance care planning process in pediatric patients with life limiting conditions irrespective of the families’ geographical background. Variations regarding MOLST rather reflect the high heterogeneity regarding norms, values, and attitudes towards ACP in children within geographical groups than systematic difference between geographical groups. Our data suggests that other sources of variation as the patients underlying disease might be of higher importance to explain the heterogeneity of agreements on MOLST between families. In line, the rates of DNACPR orders in pediatric intensive care units in Saudi Arabia [19] were similar to the rates in Belgium and the Netherlands [20]. A recent study among Iranian patients with oncological diseases, their families, and their healthcare workers also found positive attitudes towards DNACPR orders, although more in professionals than in patients and families [21].

Perceived differences of health care professionals might exceed actual differences; however, perceived differences have not been systematically assessed in the current study.

### Strengths and weaknesses

The major limitation of this study is its group-based classification of the parental countries of origin. Even within German or Turkish families, families might differ widely pending on their religiousness, their affiliation to local minorities, or their acculturation level and thus, a large cultural heterogeneity even within these geographical defined groups must be assumed. In addition, due to the demographic properties of the study population, different countries of origin were subsumed into single groups. This results e.g., in analyzing families from Arab countries in one group which neglects the wide cultural differences among those countries. Families without a German, Turkish, or Arab background were subsumed in another group which comprises European, Asian, and African countries of origin without a common cultural basis. Therefore, true differences between those countries regarding parental decisions in advance care planning might have been missed.

Another limitation of the current study is its retrospective nature. Although we could include major confounders in our analysis as the patients’ age, disease group, or the primary location of care, other confounders could not be considered, e.g., the patients educational level and socio-economic status which has been shown to be associated with the attitude towards DNACPR orders in a previous study [21].

### Future research

Future studies with a larger sample should refine the analysis including other probably confounding factors as religiousness, language, the socioeconomic background, the acculturation levels of families, or their individual horizon of experience with regard to illness and death. Additionally, these studies should be accompanied by qualitative analyses in a mixed-methods fashion. Qualitative studies provide the advantage of exploring individual attitudes on the ACP process and its influence on MOLST decisions [22]. Additionally, they provide the possibility to include the children’s perspective [23]. Individualized prospective surveys seem necessary in the light of the wide range of age groups and diseases as well as sociocultural backgrounds covered in pediatric palliative care. Future studies would benefit from a more differentiated definition of the sociocultural background which should acknowledge the families’ migration history, acculturation levels, language capabilities, and religion.

## Conclusions

This study reveals that differences in MOLST decisions can vary with different geographical backgrounds of patient’s families and therefore is a step forward to reflect the sociocultural diversity of patients and their families regarding their different needs and preferences in pediatric palliative care. However, the differences found should not foster clinicians to stereotype patients and patient’s families to a culture-based “decision-making variant” [24]. Thus, individual conversations are of highest importance to support parents in their individual cultural identity and values when facing their children’s death [7]. Whether or not the differences found represent cultural preferences or barriers to appropriate palliative care due to a migration history remains unclear and should be investigated in future studies in order to improve the quality of culture-sensitive, family-centered communication skills of caregivers in pediatric palliative care. Besides the differences found and the general acceptance of the documentation of MOLST as well as the location of death did not vary between the geographical groups assessed, suggesting a broad common basis of shared norms and values. Other sources of variance as the patients’ underlying disease might have a stronger influence on advance care planning processes.

## Supplementary information

Below is the link to the electronic supplementary material.

Supplementary file1 (DOCX 27 KB)

## Data Availability

Data may be available upon request by contacting the corresponding author.

## Abbreviations

ACP: Advance care planning
DNACPR: Do not attempt cardio-pulmonary resuscitation
FC: Full code
MOLST: Medical orders for life-sustaining treatment
PPC: Pediatric palliative care
TL: Treatment limitations
