# Supplementary material for: Advance care planning and the parental geographical background in pediatric palliative home care: a retrospective chart review
Source: Eur J Pediatr. 2022 May 4;181(7):2789–97. doi: 10.1007/s00431-022-04469-w (PMC9192398; doi:10.1007/s00431-022-04469-w)
Supplement: Supplementary file 1 — Supplementary file1 (DOCX 27 KB) [file 431_2022_4469_MOESM1_ESM.docx]

**Supplemental Table 1. Distribution of the patients’ parental countries of origin.**

| **Country of Origin** | |  |  | **Number of patients** | |
| --- | --- | --- | --- | --- | --- |
|  | **Group 1: German** | | | | |
| Germany | |  |  | 181 |  |
|  | **Group 2: Turkish** | | | | |
| Turkey | |  |  | 39 |  |
|  | **Group 3: Countries with an Arab majority** | | | | |
| Iraq | |  |  | 8 |  |
| Morocco | |  |  | 5 |  |
| Syria | |  |  | 5 |  |
| Jordan | |  |  | 2 |  |
| Lebanon | |  |  | 2 |  |
| Egypt | |  |  | 1 |  |
| Palestine | |  |  | 1 |  |
| Total | |  |  | 24 |  |
|  | **Group 4: Other countries or mixed parental background** | | | | |
| Bosnia-Herzegovina | |  |  | 4 |  |
| Italy | |  |  | 4 |  |
| Greece | |  |  | 2 |  |
| Macedonia | |  |  | 2 |  |
| Pakistan | |  |  | 2 |  |
| Poland | |  |  | 2 |  |
| Romania | |  |  | 2 |  |
| Russia | |  |  | 2 |  |
| Serbia | |  |  | 2 |  |
| Sri Lanka | |  |  | 2 |  |
| Ukraine | |  |  | 2 |  |
| Afghanistan | |  |  | 1 |  |
| Albania | |  |  | 1 |  |
| Cyprus | |  |  | 1 |  |
| Eritrea | |  |  | 1 |  |
| France | |  |  | 1 |  |
| Georgia | |  |  | 1 |  |
| Hungary | |  |  | 1 |  |
| Iran | |  |  | 1 |  |
| Kazakhstan | |  |  | 1 |  |
| Kosovo | |  |  | 1 |  |
| Croatia | |  |  | 1 |  |
| Philippines | |  |  | 1 |  |
| Rwanda | |  |  | 1 |  |
| South Africa | |  |  | 1 |  |
| *Mixed (mother/father)* | |  |  |  |  |
| Thailand/Germany | |  |  | 1 |  |
| Turkey/Pakistan | |  |  | 1 |  |
| Germany/Iraq | |  |  | 1 |  |
| Germany/Montenegro | |  |  | 1 |  |
| Total | |  |  | 44 |  |
